# An empirical classification method for South Pars marls by Schmidt hammer rebound index

**DOI:** 10.1016/j.mex.2021.101366

**Published:** 2021-04-27

**Authors:** Mohammad Azarafza, Akbar Ghazifard, Fariba Asasi, Jafar Rahnamarad

**Affiliations:** a Department of Civil Engineering, University of Tabriz, Tabriz, Iran; b Department of Geology, University of Isfahan, Isfahan, Iran; c Department of Mining Engineering, Urmia University, Urmia, Iran; d Department of Geology, Zahedan Branch, Islamic Azad University, Zahedan, Iran

**Keywords:** Empirical relationship, Marls, Schmidt Hammer, Schmidt rebound index

## Abstract

The presented article provides an experimental classification for South Pars marls (SPM), southwest of Iran, using the Schmidt hammer rebound index, marl geological classes, and SPM geo-engineering characteristics. In this regard, 45 samples of marls (rock) are selected on the studied site and tested by geotechnical in-situ and laboratory tests such as Schmidt hammer, uniaxial compressive strength (UCS), laboratory direct-shear (LDS) to estimate the geo-engineering characteristics of SPM. These specimens are categorised by Pettijohn's marl classification in 3 main groups (concluded argillaceous lime, calcareous marl, and marlstone) and established the geologic class and geo-engineering properties as well as Schmidt hammer rebound index. In the meantime, the geologic classes and the Schmidt index show the logic classification. Thus, this work attempted to prepare the experimental classification based on Pettijohn's marl classification and Schmidt rebound index for SPM. According to geotechnical experiments results, the Schmidt index shows 3 main group variations like Pettijohn's marl classification.

• This method can be used to prepare the geologic status based on the Schmidt rebound index.

• This method can be useful for detailed decryption of geo-engineering characteristics of different type of marls in the studied area.

• This method can be used as a quick link for marl geologic status and geo-engineering features.

**Specifications Table**

**Table:** 

Subject Area	Earth and Planetary Sciences
More specific subject area	Rock mass classification
Method name	Empirical classification for SPM by using the Schmidt hammer rebound index
Name and reference of original method	Original method name: Schmidt hammer ASTM C805, 2018. Standard Test Method for Rebound Number of Hardened Concrete. ASTM International, West Conshohocken, PA. https://doi.org/ 10.1520/C0805_C0805M-18 ASTM D5873, 2014. Standard Test Method for Determination of Rock Hardness by Rebound Hammer Method. ASTM International, West Conshohocken, PA. https://doi.org/ 10.1520/D5873-14
Resource availability	There are no special resources and field investigation data is presented within the article.

## Method details

The Schmidt hammer (Swiss hammer), developed in the late 1940s as an indexing apparatus for non-destructive testing of concrete which has been used in rock mechanics practice since the early 1960s, mainly for estimating the uniaxial compressive strength (UCS) and Young's modulus (E_t_) of rock materials where was invented by Ernst Schmidt. Considering its long history and widespread application, the standard methods for the Schmidt hammer test have has been reformed and improved by ASTM [1,2] and ISRM [3] which might be expected to ensure consistent and reliable values and reproducible correlations for a given rock type [1]. Many published scientific reports have focused on improving data gathering procedures and developing new correlations for different rock types and geo-engineering characteristics. In Schmidt hammer index utilisation, several issues such as hammer type, rebound values normalization, specimen dimensions, surface smoothness, weathering, and moisture content, hammer orientation, data reduction, and analysis procedures continue to undermine the Schmidt hammer reliability results. In this regard, the Schmidt hammer charts are introduced to quick access to appropriate evaluations [4].

It consists of a spring-loaded mass released against a plunger when the hammer is pressed onto a hard surface. The plunger impacts the surface and the mass recoils; the rebound value is measured either by a sliding pointer or electronically. Hammer rebound readings are considered consistent and reproducible. In such fact, non-destructive tests help rock mass/intact properties evaluation and reduce the expenses for sample collection and laboratory testing. Consequently, it can determine the geo-mechanical characteristics in dense arrays of field measurements that reflect rock masses’ real inherent heterogeneity. According to the reports, there are several versions of the Schmidt hammer which is classified as N-type (used to provide data on rock types, weakness and rock compressive strengths), L-type (used to provide data on weak rocks and thin weathering crusts), and P-type (is pendulum hammer for testing materials of very low hardness). The N-type prepares data from 20 to 250 MPa. The L-type has an impact three times lower than the ‘N’ type (0.735 compared to 2.207 Nm). Also, the *P*-type prepares data of materials with compressive strengths of less than 70 kPa. When the hammer is pressed against a surface, its piston is automatically released onto the plunger. Part of the piston's impact energy is consumed by absorption (i.e., the work done in plastic deformation of the rock under the plunger tip), and is transformed into heat and sound. The remaining energy represents the impact penetration resistance (e.g., the hardness) of the surface. It enables the piston to rebound which the distance traveled by the piston after it backfires is called the rebound value (R) [4]. From a geological perspective, harder rocks have higher R values, and weak rock provides low R values representing rock masses’ geological condition. Rebound values are influenced by gravitational forces to varying degrees so that non-horizontal rebound values must be normalised regarding the horizontal direction which comprehensively described in ASTM instructions which s shown by a pointer on a scale on the side of the instrument (range 10–100). Therefore, the Schmidt Hammer must be used with care and adequately calibrated [5]. Aydin and Basu [6] suggest that the reason for force direction is that the manufacturer's correlations are derived empirically for a particular material (mostly concrete) with a relatively narrow range of mechanical properties and are often limited to two or four impact directions. The proposed alternative normalisation methods remain a wide variation in the recommended testing procedures employed by different researchers [7] particularly regarding the number of impacts used to obtain ‘R’ values. For example, the ISRM [3] recommended that one record 20 rebound values from single impacts separated by at least a plunger diameter, and average the upper ten values. Matthews and Shakesby [8] recommended 15 rebound values for each sample, with five values that deviate most from the mean being discarded. Katz et al. [9] performed 32–40 individual impacts and averaged the upper 50%. Abnormally low values are omitted for various reasons: they may relate to the fact that the rock was weakened by the hammer's actual impact on the rock surface or too small rock flaws that were not spotted visually before the effect was applied. Nevertheless, the extent of variation in the Schmidt index on rocks has led various scholars to use this index to assess the rock types (igneous, sedimentary and metamorphic) and provide a suitable variation pattern based on geological aspects. Table 1 presents several tasks utilised on different rocks, which leads to developing classification on rock mass strengths. One of these classifications was introduced by Goudie to categorise the geologic base rock types by R values [4] which is illustrated in Table 2. As can be seen from these tables’ sedimentary rocks (especially marlstones and claystones) have always had the lowest R values and wide variations, which indicate the low strength of these geo-materials.

**Table 1:** The range of UCS and R value changes in different sedimentary rocks.

Scholar	Geo-materials	Location	Schmidt index (R)	UCS (MPa)	Ref.
Day and Goudie (1977)	Hardpan limestone	England	42	67	[14]
	Dolomite	Bahrain	50	85	
	Calcareous marl	Bahrain	14.5	38	
	Limestone	Mallorca	52.7	94	
Day (1980)	Limestone (weathered to dense)	Puerta Rico	12.5 - 53.4	57.3–95	[15]
	Argillaceous lime	Yucatan	35.9	63	
	Limestone	Jamaica (Browns town)	32.1	48	
	Dolomite	Guatemala	39.7	66	
	Dolomite	Belize	39.8	66	
	Limestone	Sarawak	56.4	47	
	Limestone	Mulu	59.9	43	
	Calcareous marl	Barbados	29.8	41	
Gökçeoğlu and Aksoy (2000)	Calcareous marl	Turkey (Ankara)	27.1 - 38.6	30–40	[16]
	Marlstone	Turkey (Istanbul)	17.15 - 44.6	20–35	
Andrade et al. (2002)	Marlstone	Portugal	25	30	[17]
	Limestone	Portugal	38	55	
Hayakawa and Matsukura (2003)	Marlstone	Japan	10.5 - 32	15–25	[18]
Basarir and Karpuz (2004)	Calcareous marl	Turkey	20 - 51	33–47	[19]
	Marlstone / Claystone	Turkey	< 18	< 20	
Kahraman et al. (2004)	Calcareous marl	Turkey	35.2 - 57	27–45	[20]
Dickson et al. (2004)	Calcareous marl	Lord Howe Island	19.8 - 28.6	25	[21]
Lyew-Ayee (2004)	Marlstone	Jamaica (Montpelier)	25.5	20	[22]
	Limestone	Jamaica (Troy)	41.87 - 55.23	35–50	
	Limestone	Jamaica (Moneague)	43.67 - 44.57	40	
	Argillaceous lime	Jamaica (Chapelton)	38.04 - 38.74	35	
	Limestone	Jamaica (Somerset)	41.76	37	
Goktan and Gunes (2005)	Shalely marl	Turkey	30 - 46	25–35	[5]
Güney et al. (2005)	Limestone	Turkey (Burdur)	62 - 64	21–138	[23]
	Travertine	Turkey (Konya)	45 - 62	58–81	
	Travertine	Turkey (Bilecik)	59 - 85	6–92	
	Travertine	Turkey (Burdur- Karamanlı)	44 - 62	24–110	
	Travertine	Turkey (Antalya-Fınıke)	33	49–52	
	Travertine	Turkey (Burdur-Yeşilova)	25 - 62	91–131	
Torabi et al. (2010)	Sandstone	Iran (Coal seam)	16 - 67	25–224	[24]
	Siltstone	Iran (Coal seam)	16 - 67	25–224	
	Shale	Iran (Coal seam)	16 - 67	25–224	
	fossiliferous Shales	Iran (Coal seam)	16 - 67	25 - 224	
	Argillaceous sandstone	Iran (Coal seam)	16 - 67	25–224	
Nazir et al. (2013)	Limestone	Malaysia	28.9 - 39	52.2–85.6	[25]
Saptono et al. (2013)	Sandstone	Indonesia (Tutupan)	10 - 26	6.53–23.2	[26]
	Mudstone	Indonesia (Tutupan)	10 - 28	6.53–25.6	
Selçuk and Yabalak (2014)	Calcareous marl	Turkey (Van)	27 - 29.7	38.6–41.3	[27]
	Marlstone	Turkey (Van)	20 - 26	4.5–9.5	
	Claystone / Argillaceous marl	Turkey (Van)	10 - 22	2.5–4.5	
Kesimal and Kesimal (2015)	Limestone	Turkey (Trabzon)	37.6 - 39.5	75–120	[28]
	Sandy limestone	Turkey (Trabzon)	30.6 - 31	22.5	
	Biomicritic limestone	Turkey (Trabzon)	13.5 - 17.5	7.7–18.9	
Jobli et al. (2016)	Marlstone	Sungai Buloh	25.85	20–25	[29]
	Calcareous marl	Sungai Buloh	37.38	30–35	
	Calcareous marl	Sungai Buloh	59.51	38–43	
	Limestone	Sungai Buloh	59.2	42–46	
Azimian (2017)	Limestone	Iran (Shiraz)	59 - 22	28.7–118.4	[30]
Rajabi et al. (2017)	Limestone	Iran (Saveh)	21.3 - 29.6	33.5–42.6	[31]
Török (2018)	Oolitic limestone	Hungary (Budapest), Austria (Vienna)	37–17	18.7–35	[32]
	Porous Miocene limestone	Hungary (Budapest), Austria (Vienna), France (Loire valley)	19	22	
	Compact limestone	Hungary (Gerecse Mountains)	45	35	
	Travertine	Hungary	45–38	35 - 42	
Jamshidi et al. (2018)	Sandstone	Iran (Qom)	39–57	46.6–77.3	[33]
El Shinawi et al. (2020)	Mudstone	Egypt (May City)	31–32	34.2–43.2	[34]
	Wackestone	Egypt (May City)	24–33	33.5–41.5	
	Grainstone	Egypt (May City)	23–25	29.1–34.6	
Aly Abdelhamid et al. (2020)	Limestone	China (Hubei)	34–55.1	47.6–92	[35]

**Table 2:** The experimental rock mass classification by Schmidt index [4].

Rock classification	Schmidt index (R)	UCS (MPa)	Rock decryption
Very weak rock	10–35	1–25	Weathered sediment rocks, Marls, Claystone
Weak rock	35–40	25–50	Marlstone, Calcareous marl, Argillaceous lime
Moderate rock	40–50	50–100	Limestone, Sandstone
Strong rock	50–60	100–200	Metamorphic rocks
Very strong rock	> 60	> 200	Dense metamorphic rocks, Igneous rocks

The presented study used this idea to provide the empirical classification for marlstones’ located in South Pars (Assalouyeh) region, southwest of Iran were named SPM. The geo-engineering characteristics of South Pars are comprehensively investigated by Azarafza et al. [10], [11], [12]. This article tried to establish the link between the geo-engineering characteristics, geological origin, and Schmidt index for SPM. The SPM geotechnical properties are presented in Table 3, an estimate by geotechnical in-situ and laboratory tests such as UCS, LDS, and carbonate content. The carbonate content test results were based on Pettijohn's classification system for marls which is presented in Table 4 [13].

**Table 3:** Geomechanical properties of SPM [10].

Parameter	Number of tests	Mean	Standard Deviation
Water content (%)	45	6.123	3.061
Specific gravity (Gs)	45	2.759	1.107
γt (kN/m3)	45	22.97	1.130
γd (kN/m3)	45	21.63	1.386
Porosity (%)	45	13.82	6.060
Cohesion (kPa)	45	208.5	55.661
Friction (o)	45	25	3.991

**Table 4:** Sedimentary rocks classification by Pettijohn (1983) [13].

Categories	Classification	Main ingredients (%)	
		Carbonate	Clay
Carbonate	Lime/Limestone	95–100	0–5
	Slightly argillaceous lime	85–95	5–15
	Argillaceous lime	75–85	15–25
Marls	Calcareous marl	65–75	25–35
	Marl/marlstone	35–65	35–65
	Argillaceous marl	25–35	65–75
Clay	Calcareous mud	15–25	75–85
	Slightly argillaceous mud	5–15	85–95
	Mudstone/Claystone	0–5	95–100

**Fig. 1:**
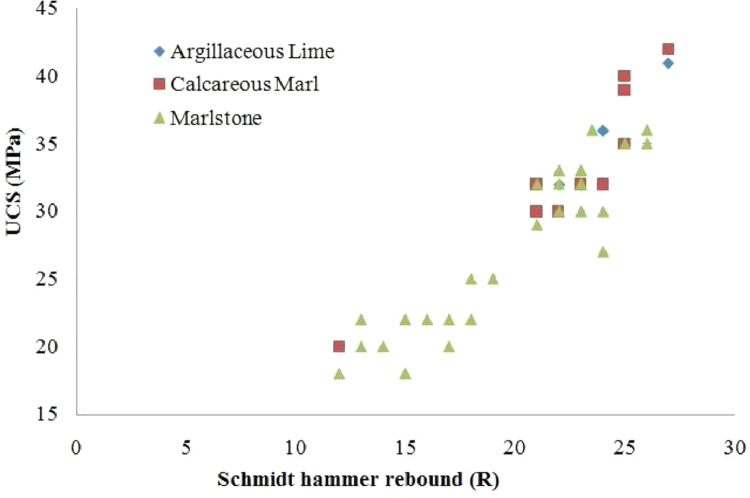
The UCS and R values variation of SPM.

**Fig. 2:**
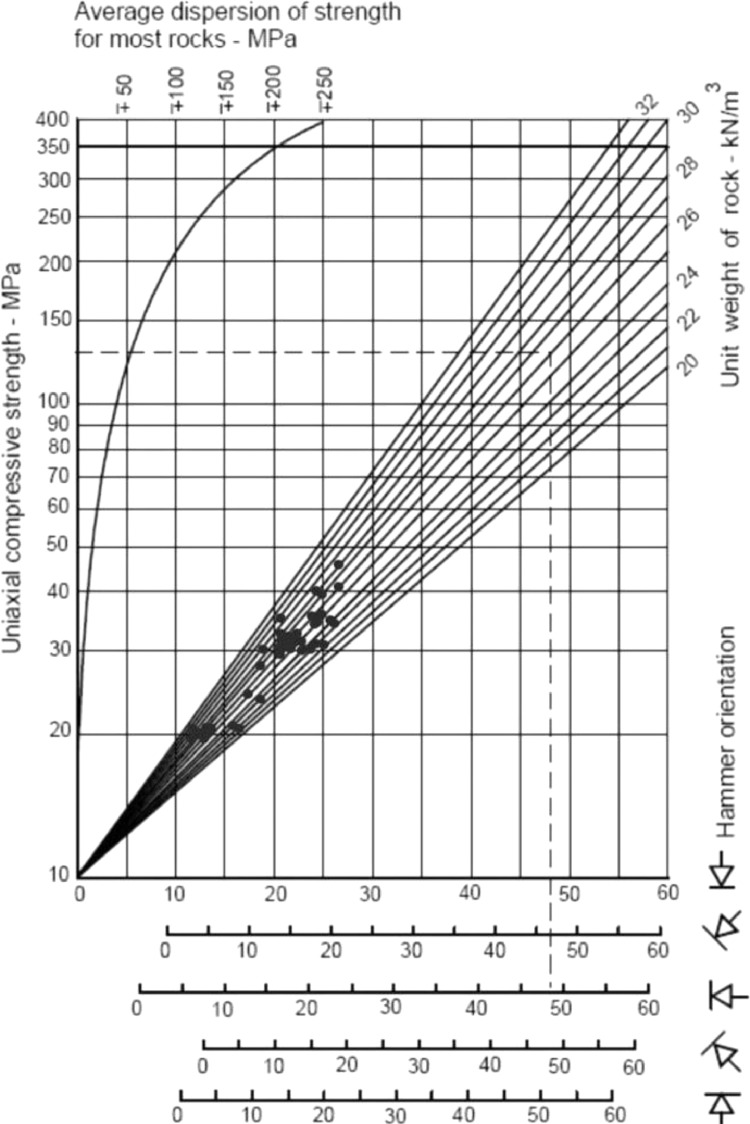
Schmidt hammer hardness index test results for SPM.

**Fig. 3:**
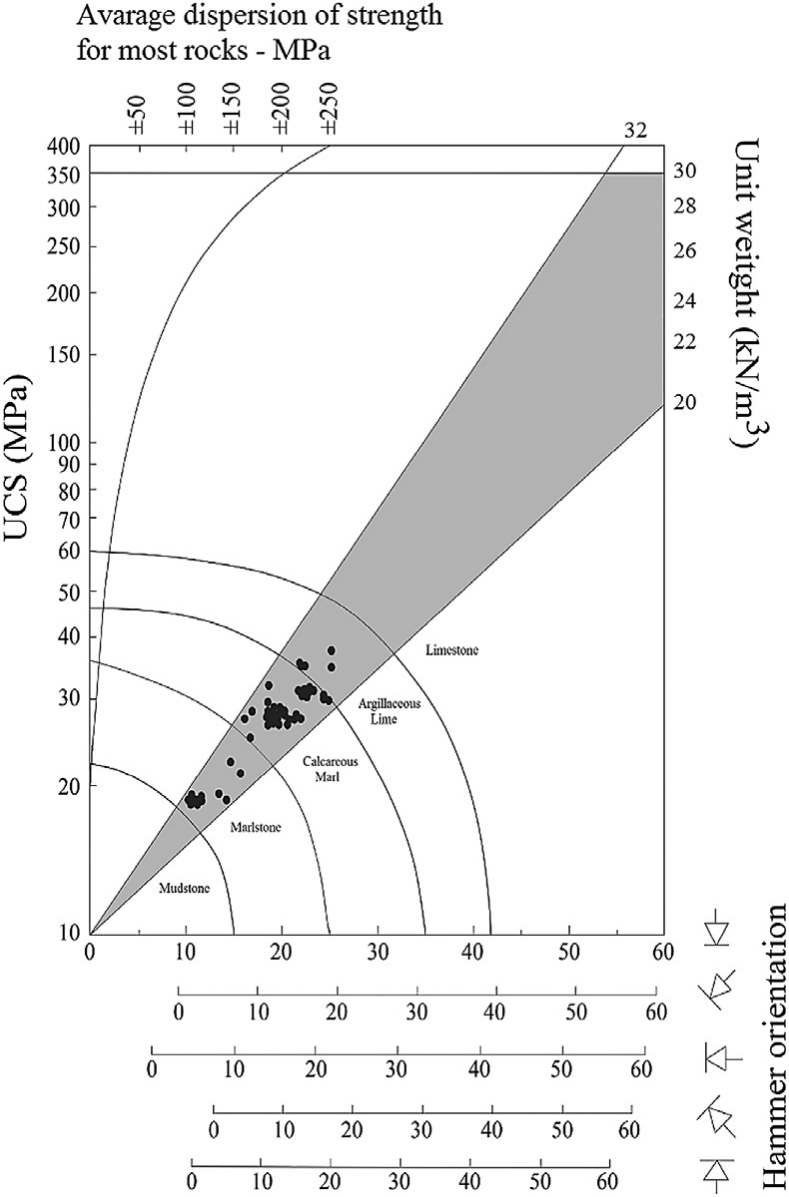
Empirical classification for SPM by using Schmidt chart.

By considering the provide data in Tables 2 to 4, the comparative Schmidt hammer test was performed on SPM samples. A hammer strike is perpendicular to the surface to provide accurate results. Then the results obtained from the experiment are recorded and plotted in the standard Schmidt chart. In this regard, the SPM samples were tested by UCS, and variations are plotted vs R-value and present in Fig. 1. Then, having information about the R and UCS values, the Schmidt chart is prepared for each of the marl categories were presented by Pettijohn. Fig. 2 is presented the results of SPM on Schmidt's chart. As shown in these figures, the results have demonstrated logical trends that explain that the Schmidt index increases almost linearly with increasing UCS in the studied region. By placing the geological type, UCS and R values in Schmidt chart can be used to separate the marls type in South Pars. Fig. 3 is presented with the SPM classification chart in the studied area. According to this figure, the carbonate content of the SPM directly controls the UCS and R (rock mass strength indicators) which can be stated that by increasing the carbonate content from Marlstone (35-65%) to Argillaceous lime (75-85%), the UCS and R will be increased.

## Declaration of Competing Interest

None.
